# A Survey of Farmers' Perceptions on Maize and Sorghum Storage Duration and Level of Pest Infestations in the Case of Two Selected Districts of Jimma Zone, Ethiopia

**DOI:** 10.1155/2021/5583387

**Published:** 2021-06-30

**Authors:** Nezif Abamecha

**Affiliations:** Department of Post-Harvest Management, College of Agriculture and Veterinary Medicine, Jimma University, Ethiopia

## Abstract

This survey study was conducted in two districts of the Jimma zone from April to July 2020 in the community of farmers who store maize and sorghum. While in Ethiopia, like in other east African countries, Maize and sorghum were an important postharvest chain for food security and income sources for small-scale rural farmers. Yet, there is a high postharvest loss of grains due to poor storage handling, storage practice, and pest infestations. And therefore, this survey study was aimed at assessing grain storage duration and level of pest infestations in selected districts of Jimma zone, Ethiopia. Two districts, Tiro Afeta and Sokoru, were selected based on the production potential of maize and sorghum grain crops. Then, three Kebeles were randomly taken from each district. Finally, two of the district's 6 Kebeles with a total of 333 households storing maize and sorghum were interviewed, while limited farmers in Tiro Afeta and Sokoru districts store sorghum 4.9% and 60.7%, respectively, for more than one year, and the current finding indicates that as long storage time, there is a prospect to be mold infestation.

## 1. Introduction

Cereal grain plays a vital role in food security and as an income source for millions of people in Ethiopia. Yet, poor storage handling and storage pests lead to high postharvest loss [1]. The reason behind such serious attack of grains in storage in Ethiopia has been reported to be the use of poor traditional storage facilities by farmers and shortage of storage technologies that allow insect pests, fungi, and other vertebrate pests to easily infest and reproduce on grain [1, 2].

Not only consuming the product, but insect pests also contaminate food gains through excretion, molting, dead bodies, and their own existence in the product, which is not commercially desirable. On the other hand, damage done by insect pests encourages infection with bacterial and fungal diseases through the transmission of their spores. They give off moisture which can cause grain moisture contents to increase enough to create a mold problem. Mold activity will in turn raise temperatures and result in an increased rate of insect reproduction [3].

Infestation of stored grains by insect pests may produce unpleasant odours, render the grain unfit for consumption, and reduce nutritional content and grain quantity thereby leading to low market prices. Furthermore, insects can facilitate entry of fungal spores into kernels or seeds by breaking the seed coat; they also disseminate fungal spores, hence potentially contributing to increased production of mycotoxins that are carcinogenic and immunosuppressive to humans. Storage grain damage and loss increase as the length of storage period increased under conduciveness of the environmental condition for the pests [4].

According to the survey study conducted by Mendesil et al. [5], storage insect pests were perceived as the major insect pests of sorghum by farmers of southwestern Ethiopia and which may also estimate to sorghum yield losses of up to 50% due to insect damage during storage while infestations of stored sorghum insect pests were common on different forms of sorghum, which are stored in various types of farm storage. Therefore, the objective of this is study was focused on assessments of farmer's perceptions on maize and sorghum storage duration and level of pest infestations in Sokoru and Tiro Afeta districts of Jimma zone, Oromia regional state, Ethiopia.

## 2. Methodology

### 2.1. Study Site Description

**Figure 1:**
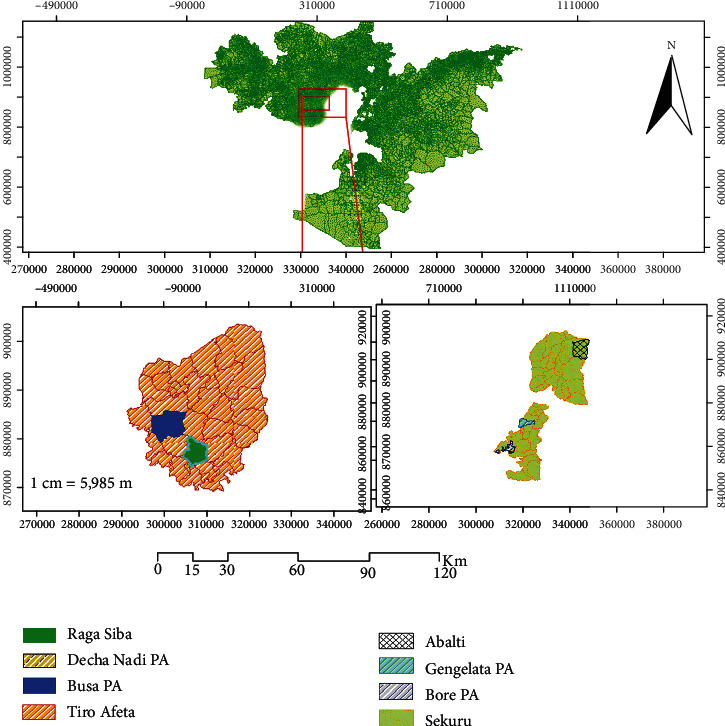
Map of the study districts.

The study was conducted in two districts of Jimma zone: Tiro Afeta and Sokoru (Figure 1) depend on their maize and sorghum production potential.

### 2.2. Sample Size and Sampling Strategy

A two-stage sampling strategy was used to select representative sample grain storing farmers. First, from both districts, three peasant associations were randomly selected as the representative of the districts. In the second stage from the list of farmer's selected Kebele numbers, households were identified using random sampling techniques. And the sample size was determined based on probability proportion using the Yamane formula [6].

(1) $$\begin{array}{c} n=\frac{N}{1+Ne^{2}}, \end{array}$$

where *N* is the size of population, *n* is the sample size, and *e* is the error of 5 percentage points.

(2) $$\begin{array}{c} n=\left.\left(\;\frac{2000}{1+2000{\left(0.05\right)}^{2}}\right.\right)=333. \end{array}$$

### 2.3. Statistical Analysis

Statistical analysis was performed using SPSS (Statistical Package for the Social Sciences) version 23.0; the data was also described in frequencies, percentages, and chi-square and *P* value.

## 3. Results and Discussion

### 3.1. Maize and Sorghum Storage Duration

The survey generally revealed that many of the farmers in the two study districts do not store maize grain for more than one year except limited farmers in the Sokoru district 12.7% (Table 1). However, few farmers in Tiro Afeta and Sokoru store sorghum 4.9% and 60.7%, respectively, for more than one year (Table 2) (Beyene and Ayelew [7]) that the majority of the farmers in Ethiopia responded to store grain products for a period of four months to one year while a small proportion of the farmers reported storing for less than 4 months and longer than one year and sorghum stores varied.

Cereal growers in the southwestern part of Ethiopia are common in other parts of the country and are predominantly smallholder farmers who keep grains for shorter durations, mainly for home consumption or to sell in a local market. However, during this storage period, stored grain is vulnerable to damage by insect pests. As a result, farmers are forced to sell their produce at very low prices immediately after harvest [8].

### 3.2. Farmers' Estimation of Pest Infestation due to Insects, Rodents, and Mold

The survey results revealed that insect, rodent, and mold infestations on farmers' maize and sorghum stores varied on the basis of storage practices. When we analyze farmer's estimations and perceptions, these stored their grains in threshed and unthreshed form, similarly in all study survey for threshed maize and sorghum are “highly attack” by insects 61.2% and 76%, respectively, than unthreshed maize and sorghum (Table 3). A similar survey was done in Ethiopia by Abamecha et al. [1] and Taddese et al. [9] which shows insect pest attack on maize considered by the greater majority of the farmers. According to Midega et al. [10], storing maize in unshelled form seemed to result in fewer pest attacks, while majority of the farmers stored their maize in shelled form. This result is also in agreement with the result of Mendesil et al. [5] which indicates, irrespective of the type of storage structure, the infestation of stored sorghum insect pests common in all types of traditional farm storages, which may be attributed to poor storage condition and lack of proper storage hygiene almost in all the traditional farm stores. However, a range of rodent management practices was being used by farmers, not just on the treatment sites that still threshed form of maize and sorghum which were highly attacked by rodent infestation 73.2% and 62.8% for both maize and sorghum in Tiro Afeta, respectively, while unshelled maize is highly attacked 54.0% by rodent infestation in Sokoru district (Table 4), although when we consider farmers' estimation level of visible mold infestation on stored maize 54.6% and 55.2% for both shelled and unshelled form in Tiro Afeta district (Table 5). This was mostly due to low yield, poor storage structures, and management as well as the situation of the farmer.

## 4. Conclusion

Most of the farmers in the survey districts stored their grain for almost more than 6 months but less than 1 year. This may be due to the fact that they rely on the lower susceptibility of maize and sorghum stored to induce pest infestation. Farmers in both the surveyed districts perceived stored grain insect pests to be the major pests of maize and sorghum which threatened grain produce. As a result, farmers can hardly store their grain free of insect pest attack as seed for the next seasons' planting, home consumption, or market. The survey findings also indicated that the majority of the farmers could store their grain from 6 months to 1-year storage duration.

## 5. Recommendation

- Farmers in the survey districts must be assisted to acquire the needed education and training on good agricultural practices particularly on early and proper harvesting and appropriate use of agrochemicals
- This would help to reduce the exposure of the dried matured grain to unfavorable weather conditions and pest infestation on the field since many of stored grain insect pest infestation arise from field contamination during harvesting and transportation to the store
- After harvesting the grain, it should be shelled, cleaned, and well dried before taken to the store for storage. These practices would help reduce the amount of insects, field moisture, and foreign materials that are transferred to the store after harvesting

## Figures and Tables

**Table 1:** Comparison of small-scale farmer's maize storage duration in study districts.

Length of storage period (month)	Tiro Afeta (*n* = 183)	Sokoru (*n* = 150)	*x*2	*P* value
Less than 3	28 (15.3)	-		
3 to 6	15 (8.2)	39 (26.0)		
6 to 9	51 (27.9)	9 (6.0)		
9 to 12	89 (48.6)	83 (55.3)		
Above 1 year	-	19 (12.7)		
			53.574^ns^	0.001

Minus sign (-) represents the data that was not taken, and values in the brackets represent % of respondents. Statistically significant at ^∗^*P* < 0.05.

**Table 2:** Comparison of small-scale farmer's sorghum storage duration in study districts.

Length of storage period (month)	Tiro Afeta (*n* = 183)	Sokoru (*n* = 150)	*x*2	*P* value
Less than 3	28 (15.3)	-		
3 to 6	13 (7.1)	10 (6.7)		
6 to 9	54 (29.5)	35 (23.3)		
9 to 12	79 (43.2)	14 (9.3)		
Above 1 year	9 (4.9)	91 (60.7)		
			136.321^ns^	0.001

Minus sign (-) represents the data that was not taken, and values in the brackets represent % of respondents. Statistically significant at ^∗^*P* < 0.05.

**Table 3:** Farmers' estimation level of insect infestations on stored grain.

Grain types and their form of storage practice	% level of insect infestation							
	Tiro Afeta (*n* = 183)				Sokoru (*n* = 150)			
	No attack	Highly attack	Moderate attack	Occasionally	No attack	Highly attack	Moderate attack	Occasionally
Maize								
Shelled	8 (4.4)	112 (61.2)	63 (19.7)	-	10 (6.7)	114 (76)	15 (10.0)	11 (7.3)
Unshelled	6 (3.3)	59 (32.2)	109 (59.6)	9 (4.9)	20 (13.3)	47 (31.3)	77 (51.3)	6 (4.0)
Sorghum								
Shelled	-	95 (52.0)	80 (43.7)	8 (4.4)	15 (10.0)	46 (30.7)	40 (26.7)	49 (32.7)
Unshelled	5 (2.7)	57 (31.1)	116 (63.4)	5 (2.7)	10 (6.7)	50 (33.3)	29 (19.3)	61 (40.7)

(-) represents none of the respondents were estimate.

**Table 4:** Farmers' estimation level of rodent's infestations on stored grain.

Grain types and their form of storage practice	% level of rodent infestation							
	Tiro Afeta (*n* = 183)				Sokoru (*n* = 150)			
	No attack	Highly attack	Moderate attack	Occasionally	No attack	Highly attack	Moderate attack	Occasionally
Maize								
Shelled	1 (0.5)	134 (73.2)	38 (20.8)	10 (5.5)	5 (3.3)	29 (19.3)	58 (38.7)	58 (38.7)
Unshelled	2 (1.1)	57 (31.1)	117 (64.0)	4 (2.2)	-	81 (54.0)	20 (13.3)	49 (39.7)
Sorghum								
Shelled	7 (3.8)	115 (62.8)	30 (16.0)	31 (17.0)	2 (1.7)	30 (20.0)	52 (34.7)	66 (44.0)
Unshelled	8 (4.4)	38 (20.8)	110 (60.1)	27 (14.8)	4 (2.7)	47 (31.3)	58 (38.7)	41 (27.3)

(-) represents none of the respondents were estimate.

**Table 5:** Farmers' estimation level of visible mold infestations on stored grain.

Grain types and their form of storage practice	% level of mold infestation/“*shagata*”							
	Tiro Afeta (*n* = 183)				Sokoru (*n* = 150)			
	No attack	Highly attack	Moderate attack	Occasionally	No attack	Highly attack	Moderate attack	Occasionally
Maize								
Shelled	63 (34.4)	17 (9.3)	100 (54.6)	3 (1.6)	31 (20.7)	5 (3.3)	68 (45.3)	46 (30.7)
Unshelled	50 (27.3)	30 (16.4)	101 (55.2)	2 (1.1)	8 (5.3)	31 (20.7)	45 (30.0)	66 (44.0)
Sorghum								
Shelled	63 (34.4)	20 (10.9)	90 (49.1)	10 (5.5)	33 (22.0)	15 (10.0)	26 (17.3)	76 (50.7)
Unshelled	68 (37.1)	16 (8.7)	70 (38.2)	29 (15.8)	4 (2.7)	79 (52.7)	21 (14.0)	46 (30.7)

## Data Availability

The data used to support the findings of this study are available from the corresponding author upon request.
